# Feasibility, reliability, and clinical validity of the Test of Attentional Performance for Children (KiTAP) in Fragile X syndrome (FXS)

**DOI:** 10.1186/1866-1955-4-2

**Published:** 2012-02-08

**Authors:** Andrew Knox, Andrea Schneider, Floridette Abucayan, Crystal Hervey, Christina Tran, David Hessl, Elizabeth Berry-Kravis

**Affiliations:** 1 Department of Pediatrics, Rush University Medical Center, 1725 West Harrison, Suite 718, Chicago, IL 60612, USA; 2 MIND Institute, University of California-Davis School of Medicine, Sacramento, CA, USA; 3 Department of Psychiatry and Behavioral Sciences, University of California-Davis School of Medicine, Sacramento, CA, USA; 4 Departments of Neurological Sciences and Biochemistry, Rush University Medical Center, 1725 West Harrison, Suite 718, Chicago, IL 60612, USA

## Abstract

**Background:**

Attention and inhibition are core executive-function deficits in FRagile X syndrome (FXS). This pilot study evaluated the feasibility, reproducibility, and clinical relevance of the KiTAP, a computer-based pictorial measure of attention and inhibition with an enchanted-castle theme, in an FXS cohort.

**Methods:**

The 8-subtest KiTAP battery (as many subtests as each could perform) was given to 36 subjects with FXS, of variable age and cognitive/behavioral functioning, and 29 were retested, with an interval of 2 to 4 weeks between sessions. Subjects were rated by parents on the Aberrant Behavior Checklist-Community Edition (ABC-C) and Behavior Assessment System for Children, Second Edition (BASC-2). Feasibility, ceiling and basal effects, and data range and distribution analyses were used to eliminate outliers and invalid data points. Reproducibility of scores was analyzed using intraclass correlation coefficients (ICCs) and validity/clinical relevance was assessed by correlating KiTAP scores with ABC-C and BASC-2 scores.

**Results:**

Most of the participants with FXS were able to complete the Alertness, Distractibility, Flexibility, and Go/NoGo subtests.About 50 to 60% completed the Visual Scanning and Vigilance subtests, and 20 to 25% completed the Sustained Attention and Divided Attention subtests. A panel of seven scores from four subtests were identified as feasible for most subjects, lacked excessive ceiling, basal, or learning effects, exhibited an acceptable range and distribution of scores, had good reproducibility (ICC > 0.7), and correlated with behavioral ratings for hyperactivity or attention (*P *< 0.01). Only minor differences in performance on the KiTAP were seen between mental age-matched cohorts of subjects with FXS and non-FXS intellectual disability.

**Conclusions:**

The KiTAP can be administered to cohorts with FXS over a wide range of function with valid reproducible scores. With additional validation, it could represent a useful outcome measure for assessment of attention/executive-function abilities in clinical trials targeted to these core deficits in FXS.

## Background

Fragile X syndrome (FXS) is the most common known inherited cause of intellectual disability (ID), learning disability, and autism, with an estimated frequency in the range of about 1:2500 to 1:4000 [1]. FXS results from a trinucleotide repeat (CGG) expansion mutation of more than 200 repeats (full mutation) in the promoter of *FMR1 *(Fragile X mental retardation 1 gene), which leads to transcriptional silencing of *FMR1*, and loss or significant reduction of expression of the gene product, the Fragile X mental retardation protein (FMRP). FMRP is an RNA-binding protein that acts as a negative modulator of dendritic translation. Loss of FMRP results in excessive and dysregulated dendritic translation, producing aberrant dendritic morphology and synaptic plasticity, and leading to abnormal development and cognition [2-4]. In addition to intellectual disability, the resultant behavioral phenotype is characterized by prominent deficits in attention and inhibitory control; autistic symptoms including social and communication deficits, and stereotypic behavior; social anxiety and withdrawal; hyperarousal; sensory defensiveness; and gaze aversion [5,6].

Recent advances in the neurobiology of FXS have suggested that many of the phenotypic features of the disorder arise from enhanced activity of translational activation pathways regulated by metabotropic glutamate receptors 1 and 5 (mGluR1, mGluR5), as a result of absence of FMRP [7]. In support of this, most known phenotypes in the mouse and fly models of FXS (which lack FMRP) can be reversed by pharmacologically [8] or genetically [9] downregulating these pathways. These groundbreaking studies have set the stage for pharmacological trials in humans with FXS, designed to target the excess activity in mGluR-regulated translational pathways in neurons [10].

Thus, there is an urgent need to develop objective and well-validated outcome measures that assay core FXS phenotypes. Boys with FXS show larger attention and executive function (EF) deficits than do mental age (MA)-matched boys with Down syndrome or typically developing boys, particularly in areas involving switching attention and inhibiting repetitious behavior [11]. Thus, EF and inhibition problems such as hyperactivity, impulsiveness, and distractibility are thought to be core features of FXS, occurring in about 80 to 90% of males and at least half of females in survey studies [12,13]. Behavior-rating scales, regarded as the standard means of assessing these symptoms, are subject to problems of rater bias and placebo effects. Continuous performance tasks (CPT) can more objectively measure EFs, and have the benefit of being responsive to relatively short-term medication treatments, making them a potentially useful type of efficacy test for early phase clinical trials and for testing response to interventions in clinic [14-16]. Efforts to use CPTs to measure function in these areas have been problematic in FXS because of the cognitive impairment and widely varying levels of function. Previous CPTs used in medication trials in FXS have been too difficult for the majority of participants (for example, the Integrated Visual and Auditory (IVA) CPT [17]) or too easy for the higher-functioning and adult participants, resulting in a high frequency of ceiling scores (for example, the North Carolina Fragile X Project CPT (FXCPT)[18] Clinical experience of patients with FXS suggests that standard CPTs used in clinical practice, such as the Connors CPT, tend to be too difficult or too long for many individuals with FXS, thus meaningful data cannot be obtained, or the individual loses interest and does not complete the task. Most CPTs used in clinical practice such as the IVA+Plus (BrainTrain, Richmond, VA, USA) are validated with a lower age limit of 5 to 6 years. Because the average MA of adult males with FXS is 5 to 6 years [19], these tasks lack the range to accurately measure the abilities of at least half of adult males with FXS and of less than half of younger males who have a lower average MA. Many of these CPTs are not interesting to the participants, presenting just simple numerals for subject responses, and are administered over a longer time period than most males with FXS can tolerate. Although the tests are designed to be uninteresting in order to test attention focusing over time in the general population, a large fraction of the FXS population, in whom ability to maintain attention and EF is much more impaired, will often discontinue the task after a short period of cooperation. Refusal to perform tasks perceived as difficult or long is a major problem in testing of individuals with FXS [20], and it is documented that boys with FXS have difficulty completing executive-function tasks [21]. By contrast, using a simplified test with limited range such as the North Carolina FX-CPT, may result in higher-functioning individuals with FXS showing only ceiling scores [21].

The Test of Attentional Performance for Children (Testbatterie zur Aufmerksamkeitsprüfung für Kinder; KiTAP) is a computer-based CPT and EF battery [22] that has not previously been used to measure cognitive function in subjects with FXS. The KiTAP was adapted for use in children from the Tests of Attentional Performance (TAP), a test used since the late 1990s to measure attention and EF performance in adults with various medical, neurological, and psychiatric conditions. KiTAP and TAP have been translated from the original German into validated versions in English, French, Spanish, and Italian [23]. The KiTAP was devised to be interesting to children, with an enchanted-castle theme as opposed to abstract stimuli, and has been used to show attentional impairment, with slower reaction times and increases in errors and omissions correlating with decrements in intelligence quotient (IQ) in liver-transplanted 6 to 12-year-old children [24], increased impulsivity (false alarms) in lead-exposed 8 to 12-year-old children that are more evident than IQ decrements [25], and improvement in Sustained Attention measures after administration of methylphenidate to 5 to 12-year-old children with attention deficit hyperactivity disorder [16]. The KiTAP is composed of eight tests that vary in length and difficulty level, thus it may be possible to use this test to measure attentional function over the broad range of intellectual ability present in a cohort with a disorder such as FXS. Other reasons for choosing the KiTAP to study FXS are the visual nature of the test, given the high preference for visual stimuli seen for subjects with FXS, and the identifiable and high-interest 'characters' presented in the enchanted-castle theme.

In this study, we sought to determine the feasibility (level of function required to achieve scores on different subtests without ceiling or basal effects), reproducibility (consistency of scores in a test/retest setting), and validity (correlation with meaningful measures of maladaptive behavior) of the subtests of the KiTAP in a population with FXS. Additionally, scores were compared with those of MA controls with ID as a means of evaluating specificity of performance patterns on the KiTAP in FXS.

## Methods

The study was approved by the institutional review board at Rush University Medical Center (RUMC), and all participants or their parents signed informed consent according to an approved protocol.

### Participants

Participants with FXS were recruited through the Fragile X Clinic and Research Program at RUMC, In total, 36 participants (28 male, 8 female; mean ± standard deviation (SD) 18.0 *± *10.24 years of age, range 7 to 50) with the full Fragile X mutation confirmed by standard testing using Southern blot combined with PCR. All participants were tested using the computer-based KiTAP test.

Subject participation was not limited by age, gender or IQ score because the intent of the study was to evaluate a wide range of subjects to determine the age and functional range over which the KiTAP could be used as an outcome measure in clinical trials with subjects with FXS. It was important to evaluate the KiTAP in both genders, and in both children and adults, given that trials of new targeted treatments for FXS would probably involve all these groups. However, all subjects tested were verbal and able to speak at least in phrases, and all had sufficient receptive language to follow basic directions.

When available, cognitive (IQ) assessments were obtained from previous tests (within the past 2 years for subjects under 15 years of age, and within the past 5 years for subjects over 15 years of age at the time of the IQ test) performed at RUMC or from psychological assessments at the participant's school. Subjects for whom IQ test results were not available were not precluded from participating in the study. IQ assessments represented scores from the Wechsler Intelligence Scale for Children (WISC), Wechsler Adult Intelligence Scale, or Stanford-Binet tests, and were available for 24 (17 males and 7 female) participants with FXS. MA was calculated from IQ using the formula

MA=IQ/100×chronologicalage,

with a maximum value of 15 used for chronological age.

The comparison cohort, consisting of 25 individuals with ID (13 male and 12 female; mean ± SD 11.4 ± 4.4 years of age. range 5 to 24), was recruited and tested using identical protocols at the Medical Investigation of Neurodevelopmental Disorders (MIND) Institute at UC Davis. This group consisted of 12 subjects with idiopathic ID, 8 with Down syndrome, 2 with fetal alcohol syndrome, 1 with 17q21.31 deletion syndrome, 1 with 22q11.2 deletion syndrome, and 1 with ID and autism. IQ measures, performed with the WISC or Stanford-Binet tests, were available for all ID participants. Although the FXS and ID subjects were tested at different sites, the methods for administration of the KiTAP were standardized as much as possible through phone conferences and a face-to-face meeting. The ID group was examined to see if there were differences in performance patterns that might be attributed to the FXS phenotype alone rather than to general ID. For this purpose, retest was not necessary, and so the ID group was not retested.

Distribution of psychotropic medication use in the FXS group was as follows: no drug (14 subjects); one drug (10 subjects), two drugs (8 subjects), and three drugs (2 subjects). Medications used were stimulants (15 subjects), selective serotonin uptake inhibitors (SSRIs)/antidepressants (10 subjects), antipsychotics (4 subjects), and lithium (1 subject).. Distribution of psychotropic medication use in the ID group was: no drug (18 subjects), one drug (3 subjects), two drugs (3 subjects), and three drugs (1 subject). Medications used were stimulants (2 subjects), SSRIs/antidepressants (2 subjects), antipsychotics (4 subjects), valproic acid (1 subject), and clonidine (2 subjects).

### KiTAP

The KiTAP is composed of eight subtests designed around a theme (an enchanted castle) specifically designed to be accessible to young children, thus providing more motivation to sustain interest in testing than CPTs and other EF batteries based on abstract symbols. Each subtest measures a different aspect of cognition: alertness, distractibility, divided attention, flexibility, reaction control (inhibition), sustained attention, vigilance, and visual scanning. The Alertness subtest (The Witch) requires subjects to tap a button every time a stimulus (a witch) appears on the screen. The Distractibility subtest (The Happy and Sad Ghosts) requires subjects to tap a button when a target stimulus (a sad ghost) appears on the screen while ignoring distracters that appear shortly before the stimulus; subjects are supposed to 'cheer up' the sad ghost by pushing the button and must not respond when the happy ghost because it does not need cheering up. The Flexibility subtest (The Dragons' House) requires subjects to alternate between identifying blue and green dragons which seem on random sides of the screen by tapping one of two buttons. The Go/NoGo or inhibition subtest (The Bat and the Cat) requires subjects to tap a button when the target stimulus (a bat) is presented, while refraining from hitting the button for the non-target stimulus (a cat). The Visual Scanning subtest (The Witches' Parade) requires subjects to scan a grid of 25 witches; subjects press one button if all witches are flying in the same direction, and hit a second button if one of the witches is flying in the opposite direction. The Vigilance subtest (The Mirror) is similar to Go-NoGo, but the target stimulus (a ghost with orange eyes) appears infrequently, and the test is much longer (approximately 10 minutes). The Sustained Attention subtest (The Ghost's Ball) features a sequence of different-colored ghosts; subjects press a button when two ghosts of the same color appear sequentially. The Divided Attention subtest (The Owl) is the only task requiring processing of an auditory stimulus in addition to visual stimuli, and requires subjects to simultaneously listen to a series of high and low owl sounds and watch for target stimuli (owls with closed eyes). Subjects must press a button either when a sound is repeated or when the target stimulus appears.

Subjects spent up to 90 minutes completing all KiTAP subtests if they were able to perform the entire test (only five subjects). Most subjects spent about 30 minutes doing the test, in which they completed 4 to 5 subtests, but were unable to focus upon, or did not understand what to do in subsequent longer subtests. Subtests were administered in the order: Alertness, Distractibility, Flexibility, Go/NoGo, Visual Scanning, Vigilance, Sustained Attention, and Divided Attention. Because the last four are much longer tasks, and based on previous experience of the length of task that typically functioning individuals with FXS can tolerate, a decision was made to begin with the subtests most likely to be completed by a wide range of participants, and to leave the most difficult and longest subtests (Sustained Attention and Divided Attention) until last. We continued to administer subtests of increasing length and difficulty until the subject refused to do any more, until it became clear to the examiner that the participant was not making any real attempt to perform the task, or until the subject was not able to show that they understood the sample pretest. Although there is the possibility that this testing order may have introduced bias into the analysis of the ability of subjects with FXS to complete the subtests, the testing order was necessary, based on initial experience with pilot subjects, as administering the longer and more difficult tests first was likely to lead to refusal, thus precluding later administration of the tests the subjects would be most likely able to complete. The order of test administration was therefore driven predominantly by the length of the task and the restrictions posed by the limited attention of subjects with FXS. Further, it is important in clinical trials or for interventions in the clinic to use the same test order before and after administration of the intervention, and the most important focus of this study was to determine the reliability of the KiTAP subtests for intervention and longitudinal studies that would involve multiple testing sessions.

During the tests, subjects were aided by a research assistant (RA). Before the start of each subtest, the RA explained the goal of the test. The subject then took a short pretest to allow their understanding to be evaluated, and if necessary repeated the pretest after further explanation, up to a maximum of three pretests. If the subject was ultimately able to perform the pretest for a given subtest correctly, and the RA perceived that the subject understood the test, the subtest was then administered. If the subject could not show that they understood what to do on the subtest, the subtest was listed as 'not feasible' for that subject. The RA also encouraged subjects to continue working, and gave positive reinforcement about performance during subtests, provided breaks between subtests when necessary, and dismissed subjects when they finished all subtests or were judged unwilling or unable to continue testing. The administration method was consistent between both centers (Rush and UC Davis), including the scripts used to explain tests to subjects and the prompting strategies. The scripts used to explain the task for the pretest were those in the KiTAP instruction manual, but these were repeated and rephrased in simpler language (same content) when subjects did not understand and were unable to perform the pretest correctly. Prompts to encourage the subjects to continue working on the task were given when they stopped paying any attention to the screen and started to do something else or were clearly no longer engaged in the testing (for example, 'Watch the computer, we have to find the witches.'). Subjects were given non-specific positive feedback for continuing to work regardless of whether they were giving accurate responses or not (for example, 'You're doing a really good job with this test today.'). Subjects were not helped with the test or told to push the button when stimuli appeared. As would be expected, the number of prompts required varied depending on the subject.

### KiTAP retest

Of the 36 subjects with FXS who were tested with the KiTAP, 29 were retested 2 to 3 weeks later by the same tester in the same setting. The other seven subjects (five male, two females; mean ± SD 18.3 ± 12.4 years of age, range 9 to 44) were not retested because of scheduling issues. No changes in psychotropic medication were allowed between testing sessions. IQ data were available for 22 (16 male and 6 female) subjects who completed retesting.

### Behavioral validation measures

At the initial session, parents of all participants, including adults who consented for themselves, filled out both the Behavior Assessment System for Children-Second Edition (BASC-2) [26] and Aberrant Behavior Checklist-Community Edition (ABC-C) [27] to allow correlation of performance on the KiTAP with ratings of each subject's hyperactivity and attentional function in daily life. The ABC-C is the most widely used scale to quantify behavioral symptoms in individuals with ID including FXS [17,28], and is considered the most valid currently available scale to assess hyperactivity and impulsive behavior for a cohort of subjects with FXS. The ABC-C is a 58-item parent- or caregiver-rated scale designed to assess adaptive and maladaptive behavior of intellectually disabled people. It is divided into five subscales: Irritability (15 items), Lethargy/Social Withdrawal (16 items), Stereotypic Behavior (7 items), Hyperactivity (16 items), and Inappropriate Speech (4 items). The BASC-2 was used to supplement the ABC-C data, as the ABC-C does not specifically cover attention. The BASC-2 is a comprehensive set of behavior-rating scales measuring degree of clinically relevant problems, including aggression, anxiety, Attention Problems, atypicality, conduct problems, hyperactivity, depression, somatization, and withdrawal. All items are rated on a three-point scale. There is a parent-rated version for children aged 6 to 11 years and adolescents aged 12 to 21 years, and a self-rated version for adults over the age of 21 years. The attention and hyperactivity clinical scales were chosen for analysis as they were deemed most relevant to the dimensions of Attention and inhibitory control on the KiTAP. The Adaptability scale was also chosen for analysis, as it appeared most likely to show association with the flexibility construct of the KiTAP. There are no scales addressing attention that have been specifically validated for FXS. The BASC-2 was chosen because it has been extensively used to rate attention and hyperactivity in children with complex behavior disorders, and has been used in practice by our groups to evaluate attention and distractibility in patients with FXS, particularly for females and higher-functioning males. The children's version of the BASC-2 was used for rating individuals with FXS aged 6 to 11 years and the adolescent version for individuals aged 12 years or older, including adults (there were only three individuals in the study with FXS over the age of 22 years). The adolescent version was used for the adults with FXS (who have substantially lower MAs) because these individuals are unable to rate themselves (the adult form is self-rated), and the questions on the adolescent version were more appropriate for these individuals, whereas the items on the adult form are largely not relevant to adults with FXS.

### Data analysis

Data analysis was performed using a spreadsheet (Excel; Microsoft Corp., Redmond, WA, USA and SPSS software (IBM, Armonk, NY, USA). Several measures from each KiTAP subtest were analyzed including number correct, errors, omissions, median reaction time, and SD of median reaction time. Raw scores were used because the normalized scores on the KiTAP previously generated from a typical population of children would not be relevant to individuals with FXS. For each of these measures, the distribution of the scores was plotted, and the intraclass correlation coefficient (ICC) was computed in SPSS for measures that had an acceptable distribution (normal distribution, and no ceiling, floor, or learning effects) after outliers and non-valid data points (subject not really participating) were eliminated. To examine clinical relevance, Pearson correlations between KiTAP scores and eight behavior checklist scores (five subscale scores from the ABC-C and the three subscale T-scores from the BASC-2) were calculated. For all subjects with FXS and ID who had completed IQ testing, MA values from the assessments were used as estimates of cognitive level to allow determination of the minimum ability required for valid testing in both children and adults (for example, a given IQ in a child and adult will not represent a comparable functional level) and to allow MA-matching in the FXS/ID comparisons. Correlations between MA and scores on KiTAP measures shown to be valid and reliable were calculated. KiTAP subtest scores in FXS and ID groups were compared using the *t*-test. Because this was a pilot study, significance was set at *P *= 0.05 for all comparisons, without adjustment for multiple comparisons.

## Results

Demographic information is shown in Table 1 for both FXS and ID subjects. A wide range of chronological and MAs was represented in both the FXS and ID groups, and there was no significant difference at the initial testing session in age, gender distribution, mental age (Table 1), or KiTAP subtest performance between the total FXS cohort and the group that was retested. The ID group was significantly younger chronologically than the FXS group (*P *= 0.01 for age comparison with both total FXS cohort and retested FXS cohort).

**Table 1:** Demographic data for subject cohorts^a^

	FXS				ID cohort (n = 25)	
	Total cohort^b^		Retested cohort^c^			
		
	Male	Female	Male	Female	Male	Female
Chronological age, years						
5 to 10	6	2	5	2	5	5
11 to 20	14	3	11	2	6	7
21+	9	2	8	1	2	0
Mean *± *SD	18.2 *± *10.03	17.5 *± *11.65	19.48 *± *10.77	13 *± *5.3	13.0 *± *6.02	11 *± *4.03
Range	7 to 50	9 to 44	7 to 50	9 to 21	5 to 24	5 to 18
Mental age, years^d^						
2 to 4.99	2	0	2	0	2	3
5 to 6.99	5	2	4	2	5	2
7 to 11	10	5	10	4	6	7
Mean *± *SD	6.6 *± *1.31	7.7 *± *1.18	6.6 *± *1.4	7.7 *± *1.3	7.6 *± *3.16	6.8 *± *2.92
Range	3.6 to 8.4	6.5 to 10.1	3.6-8.4	6.5 to 10.1	3.5 to 13.8	2.15 to 11.7

^a^Abbreviations: FXS, Fragile X syndrome; ID, intellectual disability; KiTAP, Test of Attentional Performance for Children.

^b^n = 36 and 24, respectively for chronological age and IQ.

^c^n = 29 and 22, respectively for chronological age and IQ.

^d^For subjects with IQ scores available.

In the feasibility analysis for the FXS group (Table 2), the Attention, Distractibility, Go/NoGo, and Flexibility subtests were all completed by the vast majority (> 90%) of the participants. A little over half of the subjects were able to complete the Vigilance and Visual Scanning subtests. The Sustained Attention and Divided Attention subtests could be performed only by eight and five of the subjects, respectively; all these were subjects who were high functioning and had clearly performed well on the previous six subtests. The majority of subjects with FXS who completed a subtest during the first round of testing were again able to complete the same subtest when retested. The only exceptions to this were two subjects who completed the Distractibility subtest and one subject who completed the Visual Scanning subtest at the first testing session, but not in the retest. No subjects were able to perform the subtests in the retest if they had been unable to do so in the initial testing session.

**Table 2:** Feasibility of KiTAP subtests in FXS cohort and MA for ceiling and basal effects^a^

Subtest	Performed test^b^		Failed retest, n	Performed test/retest, %	MA, years (IQ, points)^c^			
				
	n	%			Some fail	All fail	Some ceiling	Most ceiling
Alertness	36	100	0	100	NA	NA	> 6.3 (45)	> 7.8 (52)
Distractibility	35	97	2	92	< 4.7 (52)	NA	> 7.1 (47)	NA
Go-NoGo	35	97	0	97	< 3.6 (45)	NA	> 7.0 (47)	NA
Flexibility^d^	33	92	0	92		NA	> 8.4 (60)	NA
Vigilance	22	61	0	61	< 7.5 (50)	< 5.4 (36)	> 7.13 (47)	> 8.1 (54)
Visual Scanning	20	56	1	53	< 7.5 (50)	< 5.4 (36)	NA	NA
Sustained Attention	8	22	0	22	< 8.2 (55)	< 7.1 (47)	NA	NA
Divided Attention	5	14	0	14	< 8.2 (55)	< 7.1 (47)	> 8.4 (60)	NA

^a^Abbreviations: FXS, Fragile X syndrome; IQ, intelligence quotient; KiTAP, Test of Attentional Performance for Children; MA, mental age; NA, not applicable

^b^Out of 36 participants.

^c^IQs are also given for reference but as the subjects vary in age, these are less refective of the ability level required to do the task than the MA.

^d^The mental age at which some subjects fail could not be derived for flexibility as IQ measures were not available for the three subjects who failed; however, subjects with MA of 3.6, 4.7 and 5.4 years were successful in completing this task both times.

Basal and ceiling effects were related to MA for the subjects with FXS for whom we had valid IQ scores (Table 2). Basal effects resulted predominantly from unwillingness to complete the test or inability to engage in the test with meaningful effort, and a ceiling effect resulted from perfect scores in number correct, errors, or omissions. The Alertness subtest was completed by all participants, but ceiling effects were found for all measures (aside from reaction time) for some subjects with MA above 6.3 years and for all subjects with MA above 7.8 years. For the Distractibility and Go/NoGo subtests, some failures were found for subjects with MA below 5.4 years, and a ceiling effect for some subjects with MA above 7.1 and 7.0 years, respectively. Flexibility was completed by all subjects whose MA was known, and did not show a consistent ceiling effect with MA. For the Vigilance and Visual Scanning subtests, failures were found when MA was lower than 7.5 years. There was also a ceiling effect for Vigilance when MA was higher than 7.13 years, but no ceiling effect for Visual Scanning. The Sustained Attention and Divided Attention subtest could not be completed by the majority of the subjects, but no ceiling effects were seen for subjects who did attempt the tests.

ICCs were calculated for measures that did not show significant basal, ceiling, or learning effects, using data from subjects who successfully completed both trials (Table 3). After applying these criteria, eight measures emerged with ICC of less than 0.6: Alertness reaction time and SD of reaction time, Distractibility errors, Go/NoGo errors and reaction time, Flexibility errors and reaction time, and Vigilance reaction time.

**Table 3:** Reproducibility of KiTAP measures with analysis of range and distribution of scores in FXS cohort^a^

KiTAP	Measure	Ceiling effects, n	Range and distribution	Revised ICC^b^
Alertness (n = 29)	Correct	17	Too many with ceiling	
	Omissions	19		
	Anticipations	9		
	RT		Good	0.87
	SD RT		Good	0.89

Distractibility (n = 26)	Correct	4		
	Omissions	1	Fair	0.56
	Errors	4	Good	0.62
	RT		Skewed	0.89 (0.95 without outlier)

Go-Nogo (n = 28)	Correct	7		
	Errors	6	Good	0.88
	Omissions	7	Good	0.57
	RT		Good	0.88
	SD RT			

Flexibility (n = 27)	Correct	0		
	Errors	1	Good	0.73
	RT		Fair	0.78
	SD RT			

Vigilance (n = 18)	Correct	6 (trial 1);10 (trial 2)	Too much ceiling, strong learning effect	
	Omissions	6 (trial 1); 10 (trial 2)		
	Errors	5 (trial 1); 13 (trial 2)		
	RT		Good but had outliers	0.71 without outlier

Visual Scanning (n = 16)	Correct	0	Too few could do test to get distribution	
	Omissions	0		
	RT			
	SD RT			

Sustained Attention (n = 8)	Correct	0	Too few could do test to get distribution	
	Omissions	0		
	Errors	0		

Divided Attention (n = 5)	Correct	0	Too few could do test to get distribution	
	Omissions	1		
	Errors	1		
	RT			

^a^Abbreviations: FXS, Fragile X syndrome; KiTAP, Test of Attentional Performance for Children; RT, reaction time; SD, standard deviation

Performed using SPSS software.

Pearson correlations between KiTAP subtest scores and clinical ratings of behavior as measured by the ABC-C and BASC-2 are shown in Table 4. In general, measures of omission correlated poorly with reported behavior in FXS; reaction time and commission errors tended to correlate better. Of the measures with good reproducibility, identified in the analyses above (Table 3), only the Go-NoGo reaction time had no significant correlation with clinical behavior; all other measures that showed good reproducibility also correlated with at least one measure of behavior. The specific behavioral correlates most commonly seen were with Hyperactivity and Inappropriate Speech on the ABC-C, and Attention Problems, Hyperactivity, and Adaptability on the BASC-2. In general, these correlates appeared meaningful based on target KiTAP measures and were in the expected direction (for example, more Go/NoGo errors correlated with higher ABC-C and BASC-2 Hyperactivity, more Flexibility errors with higher BASC-2 Attention Problems and worse Adaptability, and more Distractibility errors with higher ABC-C Hyperactivity and BASC-2 Attention Problems). A summary of behavioral correlates with KiTAP measures showing good reproducibility is given in Table 5. Using an intercorrelation matrix for the seven KiTAP measures showing good reproducibility and validity (measures shown in Table 5), several orthogonal measures and several with moderate intercorrelation were identified (Table 6), indicating that that the measures are not likely to be assessing entirely the same construct and therefore have some independence.

**Table 4:** Correlation of KiTAP measures showing acceptable feasibility, reproducibility, range and distribution with behavioral assessments on ABC-C and BASC-2 in the FXS cohort.

	Alertness RT		Distractibility errors, median	Go/Nogo errors RT		Flexibility errors RT		Vigilance RT
	
	Median	SD	Median	Median	SD	Median	SD	Median
ABC-C								

Irritability	0.28	0.01	0.46*	-0.03	0.11	-0.30	0.41*	0.38

Lethargy	0.16	0.43*	0.06	0.06	-0.19	-0.10	0.21	0.04

Stereotypy	0.21	0.28	0.35	-0.03	0.01	-0.26	0.34*	0.60**

Hyperactivity	0.49**	0.38*	0.41*	0.33*	0.08	-0.14	0.26	0.30

Inappropriate Speech	0.35*	0.26	0.51**	-0.07	0.11	-0.23	0.29	0.64**

BASC-2-2								

Hyperactivity	0.16	0.34*	0.26	0.36*	0.07	0.20	-0.34*	0.05

Attention Problems	0.27	0.25	0.41*	0.08	-0.17	0.47**	-0.04	0.24

Adaptability	-0.41*	-0.42*	-0.40*	-0.06	-0.24	-0.34*	-0.22	-0.38

Subjects for correlations (ABC-C/BASC-2), n	27/25	27/25	23/22	26/24	26/24	27/25	27/25	18/17

^a^Abbreviations: ABC-C, Aberrant Behavior Checklist-Community Edition; BASC-2, Behavior Assessment System for Children, Second Edition; FXS, Fragile X syndrome; KiTAP, Test of Attentional Performance for Children; RT, reaction time; SD, standard deviation

*P < 0.05, **P < 0.01.

**Table 5:** Summary of acceptable measures and behavioral associations for the FXS cohort

KiTAP Measure	Correlates	
	
	ABC-C	BASC-2-2
Alertness ↑ RT with:	↑ hyperactivity**	↓ adaptability*
		
	↑ inappropriate speech*	

Alertness ↑ SD RT with:	↑ hyperactivity*	↑ hyperactivity*,
	
	↑ lethargy*	↓ adaptability*

Distractibility ↑ errors with:	↑ hyperactivity*	↑ attention problems*
	
	↑ irritability*	↓ adaptability*
		
	↑ inappropriate speech**	

Go-Nogo ↑ errors with:	↑ hyperactivity*	↑ hyperactivity*

Flexibility ↑ errors with:		↑ attention problems**
		
		↓ adaptability*

Flexibility ↑ RT with:	↑ stereotypy*	↓ hyperactivity*
		
	↑ irritability*	

Vigilance ↑ RT with:	↑ stereotypy** *	
		
	↑ inappropriate speech*	

^a^Abbreviations: ABC-C, Aberrant Behavior Checklist-Community Edition; BASC-2, Behavior Assessment System for Children, Second Edition; FXS, Fragile X syndrome; KiTAP, Test of Attentional Performance for Children; RT, reaction time; SD, standard deviation.

*P < 0.05, **P < 0.01

**Table 6:** Correlation matrix for KiTAP measures meeting the criteria of acceptable feasibility, reproducibility, range, distribution, and behavioral association.

Subtest	Measure	Alertness		Distractibility	Go-NoGo	Flexibility		Vigilance
		
		RT	SD RT^b^	Errors	Errors	Errors	RT	RT
Alertness	RT	1	0.763 (28)***	0.127 (24)	0.105 (27)	0.416 (26)*	-0.062 (26)	0.479 (17)*
	
	SD RT	-	1	-0.063 (24)	0.342 (27)	0.433 (26)*	0.094 (26)	0.228 (17)

Distractibility	Errors	-	-	1	0.535 (28)**	0.095 (25)	-0.195 (25)	0.263 (18)

Go-NoGo	Errors	-	-	-	1	0.151 (27)	-0.458 (27)*	-0.133 (18)

Flexibility	Errors	-	-	-	-	1	-0.059 (27)	0.420 (18)
	
	RT	-	-	-	-	-	1	0.231 (18)

Vigilance	RT	-	-	-	-	-	-	1

^a^Abbreviations: KiTAP, Test of Attentional Performance for Children; RT, reaction time; SD, standard deviation.

^b^Pearson correlations (*r *values) are shown with number of tests on which the correlation is based given in parentheses. *P < 0.05, **P < 0.005, ***P < 0.0001

Relationships between performance on the four KiTAP subtests that could be completed by most subjects (first testing session for subjects with FXS) and level of function were compared for the FXS and ID groups (Table 7). This analysis showed that many KiTAP measures correlated with MA in both groups, and in general, the relationships between the KiTAP measures and MA showed a similar pattern for the FXS and ID groups, suggesting a syndrome-independent general relationship between level of function and KiTAP performance. In the FXS group, performance on all measures was not significantly different in the group treated with psychotropic medication versus those who were not, with the one exception of the Distractibility errors, for which the treated group made more errors. This seems unlikely to be a direct medication effect, and may reflect a higher baseline distractibility level in those who require medication and insufficient treatment of the problem by the medication. In the ID group, medication treatment did not affect performance on any measures except Flexibility, for which there was a longer reaction time but fewer errors in the medication-treated group, consistent with either an effect of medication on performance or a characteristic of the group requiring medication. The sample size was too small to analyze for effects of individual medications or classes of medications. Additionally, 16 MA-matched individuals were chosen from each of the FXS and ID groups, with chronological age matched as closely as possible so that it was not significantly different between the two groups of (13 ± 5.7 years for the ID group and 15 ± 5.8 years for the FXS group, *P *= 0.25). Mean scores on KiTAP subtests were compared, and a significant difference was found for only two: the FXS group had fewer errors on the Distractibility (*P *= 0.053), and more errors on the Flexibility (*P *= 0.03) test.

**Table 7:** Correlation between KiTAP and MA in the FXS and ID cohorts.^a^

Test	Measure	Pearson correlations (*r *values)	
		
		FXS, n = 22	ID, n = 25
Distractibility	Errors	-0.24	-0.15
	Omissions	-0.04	-0.34*
	Median RT	0.06	-0.37*

Alertness	Median RT	-0.57**	-0.45*
	SD RT	-0.63***	-0.63***

Flexibility	Errors	-0.72***	-0.54**
	Median RT	0.18	0.00

Go/No Go	Errors	-0.40*	-0.27
	
	Omissions	-0.53**	-0.56**
	
	Median RT	-0.52**	-0.52**

^a^Abbreviations: FXS, Fragile X syndrome; KiTAP, Test of Attentional Performance for Children; ID, intellectual disability; MA, mental age; RT, reaction time; SD, standard deviation.

*P < 0.05, **P < 0.01, ***P < 0.0005

## Discussion

In this study, we sought to determine the feasibility, reliability, and validity of the KiTAP in FXS. Our data demonstrates that several measures from the KiTAP show an adequate range and good feasibility, are relatively stable over time, and are correlated with clinically meaningful behavioral constructs. The KiTAP subtests had different ranges of MA for which there were no ceiling, basal, or learning effects, and thus subjects of all MAs received a valid score on at least some of the subtests. In general, however, a significant minority of subjects with FXS could not perform the Vigilance and Visual Scanning subtests, and the large majority could not perform the Sustained Attention and Divided Attention subtests; the former because of the subjects' inability to maintain attention and motivation long enough to complete the task, and the latter because of the high level of task complexity and the subjects' inability to remember directions for both auditory and visual stimuli

Each of the subtests that were feasible for the majority of subjects with FXS had specific measures with an acceptable range and distribution of scores, and good test/retest reproducibility (ICC < 0.6). Finally, each of these feasible and reproducible measures showed some correlation with clinical behaviors, with the exception of Go-NoGo reaction time, and these correlations were mostly in the expected direction with respect to the clinical behaviors. For example, subjects who scored higher in Hyperactivity on ABC-C and BASC-2 tended to commit more impulsive errors, and subjects who scored worse on Adaptability tended to commit more errors on the Flexibility subtest. However, reaction time data was more difficult to interpret. Indeed subjects showing shorter reaction times on flexibility had higher hyperactivity ratings. This may reflect a relationship between more impulsive responding (and therefore shorter reaction time) and hyperactivity on the BASC-2, although other reaction time scores on KiTAP subtests seemed to be positively correlated with hyperactivity, suggesting that hyperactivity may result in more off-task behavior and slower reaction time. These results are difficult to reconcile, but may be just an effect of small sample size that do not reflect a clinical pattern. Our data suggest that some scores on the KiTAP may reflect the severity of aspects of the FXS phenotype, but this will require more study in a larger sample.

It is difficult to know in the FXS population whether the subtests of the KiTAP were specifically measuring the EFs they were designed to measure. Despite the correlations with the behavioral forms, which represent a crude screen for clinical relevance, there is no validated EF measure or CPT for cohorts with FXS, and therefore there is no standard for comparison to evaluate the specificity of the subtests in FXS. Previously, CPTs have been used in FXS cohorts in a clinical trial setting, and to measure medication effects on EF and attention in patients treated in clinical practice. When administered to 48 adults with FXS in a clinical trial, the IVA produced data that were impossible to analyze [17]. In this studythe combination of auditory and visual information appeared too complex for individuals with FXS to analyze, and the abstract, uninteresting nature of the stimuli failed to hold the subject's attention, resulting in random responding. The North Carolina project CPT has been subjected to some psychometric study, and was used to track development of EF and to evaluate effects of clinical stimulant treatment on attention trajectories [29] in young males with FXS, but proved to have insufficient range when used in a clinical trial with higher-functioning male and female adults with FXS, thus suggesting that it is useful only for a subset of young patients [18].

We found that many, but not all, KiTAP measures correlated with MA, both in the FXS group and the ID group. There was no association between chronological age and KiTAP performance in either group, and for the MA-matched FXS and ID groups used to compare the KiTAP subtest scores, there was no significant difference in chronological age, so age should not have been a factor limiting the comparisons. For most KiTAP measures there were no significant differences in score distributions between the two groups for the different subtests, suggesting that KiTAP gives a general measure of executive-function ability both in subjects with FXS and in those with other causes of cognitive impairment. The difference in flexibility scores, with worse performance in the FXS group, is not unexpected given that perseveration is a prominent feature of the FXS phenotype. However, it was unexpected that the FXS group made fewer errors on the Distractibility sub-test; this may be due to the high demand for rapid visual processing inherent in the test (stimuli are presented very rapidly) coupled with the relative strength in visual processing in FXS [30], or to some other property of the specific subtest, or to random chance, as the significance level was borderline.

This pilot study had a number of limitations related to the sample of subjects examined.

1) The sample size is relatively small, although the study was intended as a pilot study to help determine whether further work with the KiTAP in FXS should be pursued. Given the relative rarity of FXS, this study is similar in size to other pilot studies of measures in FXS.

2) Not all subjects provided IQ results; however, subjects with missing IQ scores were generally well distributed across KiTAP scores and age. The one exception to this rule was that the four subjects who scored poorest on KiTAP across all subtests did not have valid IQ scores. One of these subjects was probably too low functioning to complete valid IQ testing. The other subjects had very substantial clinical issues with hyperactivity and distractibility, which probably contributed to their lower scores.

3) Formal testing for autism was also not available for the majority of the subjects in the cohort, and thus the effects of autism status on performance could not be assessed; however, this analysis was outside the scope and purpose of the study in any case, and would be a topic for further research.

4) Although medication overall did not seem to have a major effect on group performance levels, it is possible that differences in the FXS and ID groups in the fraction of subjects treated with medication (the FXS group had a higher level of medication use) could have affected the comparisons between the two groups. More definitive analysis of medication effects and an understanding of the effects of individual medications on performance will require further work with a larger sample size.

5) Although females with FXS generally have a higher level of functioning than males, the number of females enrolled was insufficient to generate a sample size adequate to analyze their data separately from the males, hence we cannot be absolutely sure that the relationships between KiTAP performance and BASC-2/ABC-C scores would be the same in females and males.

6) The FXS and ID groups were tested at different sites, and despite attempts to ensure similar protocols and administration methods, differences in administration technique could have affected the results.

7) The KiTAP subtests were administered in a fixed order, which may have affected the assessment of subjects ability to perform the subtests administered later in the battery, and the examiner prompts, which were somewhat variably used across subjects, were not counted systematically for analysis of their effect on test feasibility and validity

8) The BASC-2 has not been formally normalized or evaluated in FXS, and there may be better attention measures to use for KiTAP validity analyses, although no other parent-report attention measures have been formally validated for FXS, and understanding the optimal measure for use to assess cohorts with FXS will require further study.

9) Another potential limitation of the KiTAP is the difficulty in defining a true floor for scores. Our criteria for failing a subtest only included refusal or inability to stay engaged. For a number of the subtests, subjects who are engaged in the subtest may receive a non-zero score, even if they do not understand the idea behind the test. This could lead to a situation in which a subject might have an improvement in cognitive function that was not measured by the test, if they remain beneath the threshold of actually understanding the test. However, the overall correlation of measures with MA and clinical behavior is reassuring that, in spite of this problem, KiTAP does serve as a good measure of function.

10) Lack of immediate feedback during testing may be a further limitation of the KiTAP. Because no immediate feedback about incorrect or correct responses is given by the KiTAP program during the test, subjects might become confused about the purpose of the test or disinterested in participating. The RA administering the test addressed this problem by frequently encouraging the subject to continue to participate in the task and by giving positive feedback for participation.

11) However, this strategy does have the disadvantage of adding another potential limitation: the subject performance is made somewhat dependent on the motivation and technique of the test administrator, and creates problems in interpretation of the subject's innate attention/EF skills as measured by the task. Such a limitation may be unavoidable with an FXS population because of the difficulty in getting subjects with FXS to finish the testing tasks at all without such encouragement. We attempted to at least keep the assessments consistent between subjects and trials by using the same test administrator for all trials of a given subject at each site, and creating a common protocol for administering the test. Lack of feedback about incorrect responses is likely to be helpful in that subjects do not really experience a sense of failure or frustration about their inability to perform.

12) Finally, the KiTAP castle appears on first impression to appeal more to children than to adults with FXS. Although older adult subjects may have been less engaged by the castle theme than children, this did not seem to specifically affect performance, as many adults with FXS remain interested in and enjoy cartoon characters, and some of the adults in the study expressed interest in talking about the characters after the testing session. Regardless of age, the castle 'characters' seemed to sustain attention and to be of greater interest than the abstract stimuli used in the IVA and other standard CPTs. Validated computerized EF batteries with adult visual themes are an area that could be explored in future test development and validation.

Specific measures on the KiTAP that emerged as feasible for the majority of subjects were Alertness reaction time, SD of Alertness reaction time, Distractibility errors, Go/NoGo errors, Flexibility errors, Flexibility reaction time, and Vigilance reaction time (Table 5). These measures did not produce excessive ceiling, basal, or learning effects, exhibited an acceptable range and distribution of scores, had good to excellent reproducibility, and correlated with meaningful behavior ratings. Six of these seven measures were contained in four subtests: Alertness, Distractibility, Go/NoGo, and Flexibility. In our experience administering the KiTAP, we found that these tests also have the advantage of each requiring less than 5 minutes to administer, as opposed to other subtests, which required 10 to 15 minutes. This allowed the majority of subjects to complete all four of these subtests without significant fatigue, a definite advantage for assessments involving subjects with FXS. Thus, we feel that these four subtests are best suited for use in future studies, and because this pilot study has shown that most patients with FXS can complete these subtests, the effects of test order can be further evaluated by randomizing the order of administration in future studies. Further, more work could be carried out to evaluate the characteristics of the Vigilance, Visual Scanning, Sustained Attention, and Divided Attention Subtests in a larger high-functioning FXS sample with MA of greater than 10 years to determine whether these are useful measures of response in high-functioning FXS cohorts, particularly females.

## Conclusions

In this pilot study, some measures on at least some subtests of the KiTAP showed adequate feasibility, reliability, and clinical validity for subjects with FXS across a wide range of function. This is in contrast with previous CPTs which have not allowed meaningful administration to such a range of individuals with FXS without producing substantial ceiling and floor effects [17,18]. Given the difficulties with rater bias and placebo effects that accompany behavioral rating forms addressing EF domains even with optimal administration practices, the KiTAP may be a useful objective outcome measure of attention and executive function for use in FXS clinical trials, and even to track medication responses in clinical practice. Further work is needed to evaluate the responsiveness of the KiTAP to pharmaceutical or other interventions.

## Competing interests

The authors declare that they have no competing interests.

## Authors' contributions

AK recruited and carried out testing with most of the FXS participants at RUMC, performed data and statistical analyses, and assisted with drafting the manuscript. AS assisted with data analyses. FA carried out testing with subjects at UC Davis. CH assisted with subject recruitment and carried out some testing with subjects at RUMC, and assisted with drafting the manuscript. CT assisted with recruitment and testing with subjects at RUMC. DH participated in the conception and design of the study, performed some data and statistical analyses, and assisted with drafting the manuscript. EBK conceived of, designed and coordinated the study, and participated in drafting of the manuscript. All authors read and approved the final manuscript.
